# Strongly Radioiodine-Positive Pancreatic Adenocarcinoma Mimicking Metastasis of Differentiated Thyroid Cancer

**DOI:** 10.3390/diagnostics12081934

**Published:** 2022-08-11

**Authors:** Florian Rosar, Caroline Burgard, Maximilian Linxweiler, Phillip R. Stahl, Fadi Khreish, Samer Ezziddin

**Affiliations:** 1 Department of Nuclear Medicine, Saarland University, 66421 Homburg, Germany; 2 Department of Otorhinolaryngology, Head and Neck Surgery, Saarland University, 66421 Homburg, Germany; 3 Department of Pathology, Saarland University, 66421 Homburg, Germany

**Keywords:** radioiodine therapy, iodine-131, scintigraphy, pancreatic adenocarcinoma, thyroid carcinoma

## Abstract

We present an interesting image of a strikingly intense radioiodine accumulation of a histologically proven pancreatic adenocarcinoma mimicking metastasis of differentiated thyroid cancer in a 63-year-old woman with recurrence of papillary thyroid carcinoma undergoing radioiodine therapy. This interesting image should draw attention to considering pancreatic adenocarcinoma in radioiodine-positive pancreatic lesions.

A 63-year-old woman with a history of papillary thyroid carcinoma (PTC) treated with thyroidectomy and ablative radioiodine (RAI) therapy presented with local recurrence and ipsilateral cervical lymph node metastases of PTC. After resection of these tumor manifestations, the patient underwent subsequent radioiodine (RAI) therapy with 3.8 GBq ^131^I. Whole-body scintigraphy revealed a suspicious and intense RAI-positive abdominal lesion (Figure 1A), while no pathological uptake was observed in residual cervical lymph nodes suspicious for metastases on ultrasound. Complementary [^18^F]FDG PET/CT (Figure 1B,C) and contrast-enhanced CT (Figure 1D) showed a corresponding mass in the pancreatic head with intense glucose metabolism, irregular margin and decreased contrast enhancement, respectively, suspicious for a malignant pancreatic tumor. In addition, intense [^18^F]FDG uptake was seen in cervical lymph nodes on the left side, considered as RAI-negative lymph node metastases. After completion of neck dissection with histopathological confirmation of PTC metastases, we tried to clarify the pancreatic mass using fine needle aspiration, but without representative results. The CA-19-9 serum concentration of >2000 IU/mL was highly elevated. An interdisciplinary tumor board recommended the Whipple procedure, which was subsequently performed. On histopathological examination (Figure 2), the lesion was cytokeratin 7 (CK7)-positive, thyroglobulin-negative, thyroid transcription factor-1(TTF1)-negative and classified as pancreatic adenocarcinoma.

For decades, RAI therapy has been an integral part of the treatment of patients with differentiated thyroid cancer. RAI is used both for the ablation of remnant tissue and to detect or treat RAI-positive metastases [1,2]. The key mechanism of using RAI is the iodine uptake of thyroid tissue by the sodium-iodide symporter (NIS), whose expression is mainly retained in well-differentiated thyroid cancer cells [3]. Some extrathyroidal tissues such as the salivary glands and the stomach also show physiological uptake due to NIS expression [4]. Unexpected RAI uptake has been found in benign lesions and also in malignant non-thyroid neoplasms, e.g., lung cancer, breast cancer and other malignancies [5,6,7]. It is presumed that RAI-positivity results from the functional expression of NIS and tumoral inflammatory changes. We assume that this is also the explanation in our case of RAI-positive pancreatic adenocarcinoma. A possible differential diagnosis would also have been a pancreatic metastasis of a PTC, which seems to be very rare [8,9]. Clear differentiation of pancreatic adenocarcinoma from pancreatic metastases of thyroid cancer appears to be challenging on imaging. CT signs such as irregular boundary or decreased contrast enhancement seem to be more indicative of pancreatic adenocarcinoma [10,11].

This interesting image should draw attention to considering pancreatic adenocarcinoma in RAI-positive pancreatic lesions.

## Figures and Tables

**Figure 1:**
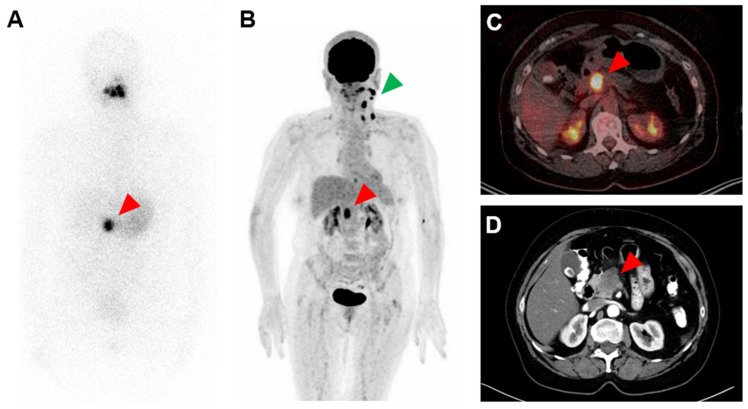
Strongly radioiodine (RAI)-positive and [^18^F]FDG-positive pancreatic adenocarcinoma. (**A**): ^131^I whole-body scintigraphy after administration of 3.8 GBq ^131^I, (**B**): MIP (maximum intensity projection) of [^18^F]FDG PET, (**C**): transversal slice of [^18^F]FDG PET/CT fusion and (**D**): transversal slice of contrast-enhanced CT. Red arrows point to RAI-positive and [^18^F]FDG-positive pancreatic adenocarcinoma; green arrow points to RAI-negative and [^18^F]FDG-positive lymph nodes metastases of papillary thyroid carcinoma in the left neck.

**Figure 2:**
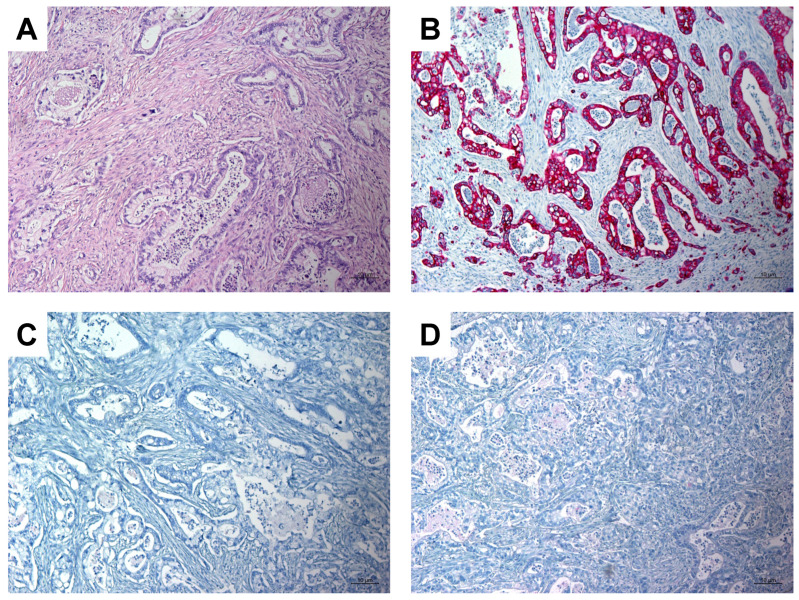
Histopathologic images showing pancreatic adenocarcinoma. (**A**): Hematoxilin-Eosin (H&E) stain, magnification 100×; (**B**): Immunohistochemistry: tumor cells show strong positive staining for CK7, magnification 100×; (**C**): Immunohistochemistry: tumor cells are negative for thyroglobulin, magnification 100×; (**D**): Immunohistochemistry: tumor cells are negative for TTF1, magnification 100×.

## Data Availability

The datasets used and analyzed in this paper are available from the corresponding author on reasonable request.
